# Novel gene therapy for rheumatoid arthritis with single local injection: adeno-associated virus-mediated delivery of A20/TNFAIP3

**DOI:** 10.1186/s40779-022-00393-0

**Published:** 2022-06-21

**Authors:** Qin Zhang, Fang-Xing Yu, Yang-Lin Wu, Cheng-Yuan Yang, Nai-Cheng Liu, Xu Zhu, Pi-Ming Zhao, Zhong-Ya Wang, Jun Lin

**Affiliations:** 1 Department of Orthopaedics, Suzhou Dushu Lake Hospital, Dushu Lake Hospital Affiliated to Soochow University, Medical Center of Soochow University, Suzhou, 215001 Jiangsu China; 2 Department of Orthopaedics, the First Affiliated Hospital of Soochow University, Soochow University, Suzhou, 215006 Jiangsu China; 3 Department of Gene Therapy, CureGenetics Co., Ltd., Suzhou, 215021 Jiangsu China

**Keywords:** Adeno-associated virus, Gene therapy, Rheumatoid arthritis, TNF-α-induced protein 3 (A20)

Dear Editor,

Multiple experiments have established TNF alpha-induced protein 3 (A20/TNFAIP3) as a critical regulator associated with rheumatoid arthritis (RA) [1, 2]. The lack of TNF-α-induced protein 3 (A20) promotes the NOD-like receptor protein 3 (NLRP3) inflammasome and induces spontaneous arthritis, while increase of A20 reduces the secretion of IL-1β and favors immunological tolerance [3, 4]. Hence, we investigate the feasibility of recombinant adeno-associated virus 6 (rAAV6)-mediated A20 gene therapy in a collagen-induced arthritis (CIA) model.

We first investigated cytomegalovirus (CMV) promoter-regulated gene delivery (Additional file 1: Fig. S1). rAAV6 (Additional file 1: Fig. S1a, b) was transfected into 293 T cells (Additional file 1: Fig. S2a) to enhance A20 expression (Additional file 1: Fig. S2b). A 10 μl of volume rAAV6-CMV-A20 containing 1 × 10^12^ viral genomes (vg) was injected into the left knee, ankle, and tarsal area of CIA mice, while the same dose of rAAV6-CMV-EGFP was injected into the right side (Additional file 1: Fig. S2c). rAAV6 was widely distributed in various cell types (Additional file 1: Fig. S2d) and significantly enhanced A20 expression until 5 weeks after injection (Fig. 1a). Notably, rAAV6-CMV-A20 decreased the clinical arthritis score, paw thickness and total porosity (*P* < 0.001), increased the bone volume-to-tissue volume ratio (BV/TV, *P* < 0.001), trabecular number (Tb.N, *P* = 0.008), and trabecular thickness (Tb.Th, *P* = 0.002) (Fig. 1b–d, Additional file 1: Fig. S2e–j), and suppressed pannus formation, bone erosion, and cartilage destruction (Fig. 1e). We also found that rAAV6-CMV-A20 significantly suppressed the expression of NLRP3, caspase-1, and IL-1β (Additional file 1: Fig. S2k–m). Thus, the results verified our hypothesis that rAAV6-mediated A20 overexpression is an effective RA therapy.

**Fig. 1:**
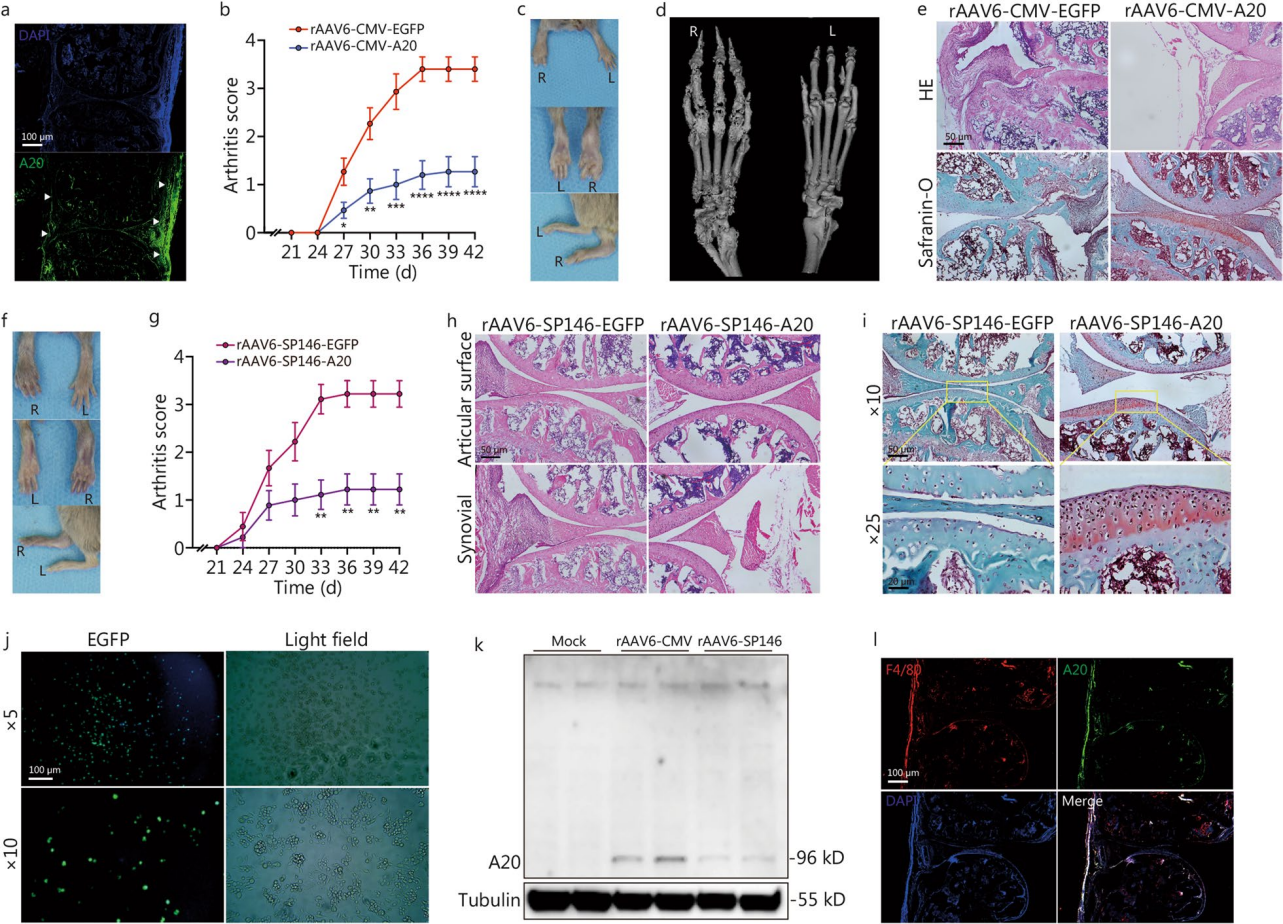
rAAV6-CMV-A20 inhibits inflammation in CIA mice. **a** Immunofluorescence staining of A20 in joints that were injected with rAAV6-CMV-A20 (white arrow: synovium). **b** Clinical arthritis score of hind paws injected with rAAV6-CMV-EGFP or rAAV6-CMV-A20 (*n* = 15); Statistical analyses were performed between hind paws injected with rAAV6-CMV-EGFP and rAAV6-CMV-A20 using paired *t*-tests. **c** Hind paws of CIA mice injected with rAAV6-CMV-EGFP (right) or rAAV6-CMV-A20 (left) on the day 42nd after primary immunization. **d** Micro-CT 3D reconstruction image of hind paws. **e** HE or Safranin-O staining of joint specimens injected with rAAV6-CMV-EGFP or rAAV6-CMV-A20. **f** Hind paws of CIA mice injected with rAAV6-SP146-EGFP (right) or rAAV6-SP146-A20 (left) on the day 42nd after primary immunization. **g** Clinical arthritis score of hind paws injected with rAAV6-SP146-EGFP or rAAV6-SP146-A20 (*n* = 9); Statistical analyses were performed between hind paws injected with rAAV6-SP146-EGFP and rAAV6-SP146-A20 using paired *t*-tests. HE (**h**) and Safranin-O (**i**) staining of joint specimens injected with rAAV6-SP146-EGFP or rAAV6-SP146-A20. **j** Fluorescence image of RAW264.7 cells transfected with rAAV6-SP146-EGFP. **k** Western blotting of A20 in RAW264.7 cell lysates following transfection with rAAV6-CMV-A20 or rAAV6-SP146-A20. **l** Immunofluorescence staining of A20 and F4/80 in joint specimens injected with rAAV6-SP146-A20. CMV cytomegalovirus, NLRP3 NOD-like receptor protein 3

The CMV promoter may attract safety concerns. Therefore, considering that macrophages and fibroblast-like synoviocytes account for the primary components of synovial tissues, especially in RA, we tested which cell type predominantly overexpressed A20. We constructed rAAV6-COL1α-A20 (Additional file 1: Fig. S1c) targeting fibroblast-like synoviocytes and rAAV6-SP146-A20 (Additional file 1: Fig. S1e) targeting macrophages [5]. A 10 μl volume of rAAV6-COL1α-A20 or rAAV6-SP146-A20 (1 × 10^12^ vg) was injected into the left knee, ankle, and tarsal area, while the same dose of rAAV6-COL1α-EGFP (Additional file 1: Fig. S1d) or rAAV6-SP146-EGFP (Additional file 1: Fig. S1f) was injected into the same areas on the right side (Additional file 1: Fig. S3a). In contrast to rAAV6-COL1α-EGFP, rAAV6-COL1α-A20 exhibited nearly no effect on suppressing RA symptoms. However, rAAV6-SP146-A20 showed anti-rheumatic therapeutic effects compared with rAAV6-SP146-EGFP. Similar to rAAV6-CMV-A20, rAAV6-SP146-A20 reduced the clinical arthritis score, paw thickness (Fig. 1f, g; Additional file 1: Fig. S3b, c), total porosity (Additional file 1: Fig. S3d), bone erosion activity (Additional file 1: Fig. S3e–g), pannus formation, and cartilage destruction (Fig. 1h, i). rAAV6-SP146-EGFP was well transfected into RAW264.7 cells (Fig. 1j) and exhibited dominant distribution in macrophages (Additional file 1: Fig. S3h). rAAV6-SP146-A20 significantly enhanced A20 expression (Fig. 1k, l) and the collective results further indicated the therapeutic benefits of rAAV6-mediated A20 overexpression. Importantly, macrophages were found to be responsible for the rAAV6-mediated A20 overexpression.

The anti-rheumatic benefits of rAAV6-SP146-A20 were further verified by simultaneous injection of rAAV. CIA mice were injected with rAAV6-SP146-EGFP or rAAV6-SP146-A20 on both sides (1 × 10^12^ vg, Additional file 1: Fig. S3i). As expected, rAAV6-SP146-A20 significantly relieved the arthritis symptoms (Additional file 1: Fig. S3j–o, Fig. S4a–c). Furthermore, we tested whether the therapeutic effect was dependent on viral genomes. CIA mice were injected with rAAV6-SP146-EGFP at a dose of 1 × 10^12^ vg (EGFP) or rAAV6-SP146-A20 at a dose of 1 × 10^8^ vg (E8), 1 × 10^10^ vg (E10), or 1 × 10^12^ vg (E12, Additional file 1: Fig. S3p). The results revealed that the protective effect of rAAV6-SP146-A20 was dose-dependent. The E12 group exhibited the lowest clinical arthritis score and increases in paw thickness, bone erosion activity, pannus formation, and cartilage destruction (Additional file 1: Figs. S3q–v, S4d–e). As the treatment dose increased, so did the expression of A20, while NLRP3, caspase-1, and IL-1β were gradually inhibited (Additional file 1: Fig. S4f–g).

Our hypothesis that A20 overexpression is a reasonable strategy for the treatment of RA was initially evidenced. We not only demonstrated the therapeutic effects against RA of ubiquitous CMV promoter-driven delivery of A20, but more importantly, we identified the key role of macrophage-like synovial cells in responding to rAAV6-mediated gene delivery of A20.

## Supplementary Information

**Additional file 1**. Materials and methods. **Fig. S1:** Construction of rAAV expression plasmids. **Fig. S2:** Therapeutic effect of rAAV-CMV-A20. **Fig. S3:** Verification of the therapeutic effect of rAAV6-SP146-A20. **Fig. S4:** rAAV6-SP146-A20 inhibits NLRP3-mediated inflammation in CIA mice.

## Data Availability

All data needed to evaluate the conclusions in the paper are present in the paper or the Additional files. The data are available from the corresponding author on reasonable request.

## Abbreviations

A20/TNFAIP3: TNF-α-induced protein 3
BV/TV: Bone volume-to-tissue volume ratio
CMV: Cytomegalovirus
CIA: Collagen-induced arthritis
NLRP3: NOD-like receptor protein 3
RA: Rheumatoid arthritis
Tb.N: Trabecular number
Tb.Th: Trabecular thickness
