# Supplementary material for: Novel gene therapy for rheumatoid arthritis with single local injection: adeno-associated virus-mediated delivery of A20/TNFAIP3
Source: Mil Med Res. 2022 Jun 21;9:34. doi: 10.1186/s40779-022-00393-0 (PMC9210758; doi:10.1186/s40779-022-00393-0)
Supplement: Supplementary file 1 — Additional file 1. Materials and methods. Fig. S1: Construction of rAAV expression plasmids. Fig. S2: Therapeutic effect of rAAV-CMV-A20. Fig. S3: Verification of the therapeutic effect of rAAV6-SP146-A20. Fig. S4: rAAV6-SP146-A20 inhibits NLRP3-mediated inflammation in CIA mice. [file 40779_2022_393_MOESM1_ESM.pdf]

Materials and methods

Animal experiment

DBA/1 male mice, aged 8 weeks, were purchased from Beijing Charles River Company (Beijing, China) and housed under pathogen-free conditions with adequate food and water in a SPF animal laboratory. All animal experiments were conducted with the permission of the Ethical Committees on Laboratory Animal Welfare of Soochow University, Suzhou, China. The mice were anesthetized by isoflurane, and rAAV6 was articular-injected using a Hamilton injection syringe.

Collagen-induced arthritis model

The CIA mouse model was established by double immunization. For the first immunization, mice were injected subcutaneously at the end of the tail with an emulsion of equal volume of bovine type-II collagen solution (2 mg/ml) and complete Freund’s adjuvant (4 mg/ml). Twenty-one days after the first immunization, the booster immunization was administered with bovine type-II collagen solution emulsified in incomplete Freund’s adjuvant.

Clinical scoring

The mice were scored once three days after the second immunization. A score of 0–4 was assigned to each hind paw as follows: 0, normal; 1, swelling of the toes; 2, swelling of the ankle and/or tarsus; 3, moderate swelling of the ankle and/or tarsus, or mild swelling of both; and 4, severe swelling of the entire paw.

Micro-CT analysis

Hind paws collected from every group were subjected to micro-computed tomography (Micro-CT) scanning (SkyScan 1176, Aartselaar, Belgium). The parameters of the X-ray were set at a current of 500 µA with a voltage of 50 kV. The scanning per layer was 9 µm. CTAn software was used to analyze the hind paws and calculate the bone volume-to-tissue volume ratio (BV/TV, %), trabecula number (Tb.N, mm^{-1}), trabecula thickness (Tb.Th, mm), and total porosity (%).

Histology and immunofluorescence staining

For the observation of EGFP, the mouse knee joints that underwent articular injection with rAAV6-CMV-EGFP or rAAV6-CMV-A20 were embedded in optimal cutting temperature compound (OCT). The frozen specimen was then cut into frozen slices of 6 µm thickness. The slices were incubated with

F4/80 (rabbit, Abcam, U.S.) overnight at 4°C. Following washing three times with PBS, slices were incubated with Goat Anti-Rabbit IgG H&L (Alexa Fluor®647, Abcam) or DAPI (Keygen BioTECH, China). To evaluate the severity of inflammation, the mouse knee joints were embedded in paraffin. The specimens were cut into slices of 6 μm thickness. HE and Safranin-O staining was then performed following the manufacturer's instructions.

For immunofluorescence assays, knee joint sections were incubated with A20 (mouse, R&D), NLRP3 (rabbit, Proteintech), caspase-1(rabbit, Proteintech), IL-1β (rabbit, Proteintech), or F4/80 (rabbit, Abcam) overnight at 4°C. Following washing three times with PBS, slices were incubated with Goat Anti-Mouse IgG H&L (Alexa Fluor®488, Abcam) and Goat Anti-Rabbit IgG H&L (Alexa Fluor® 647, Abcam) for 60 min, and then the slices were incubated with DAPI (Keygen BioTECH, China) for 10 min.

Statistical analysis

Unless otherwise stated, comparisons between two conditions were performed via the two-tailed Wilcoxon matched pairs test, while comparisons between two groups of mice over time were performed using the two-tailed Mann-Whitney *U* test. Statistical analyses were performed using GraphPad Prism software. A *P*-value < 0.05 was considered statistically significant.

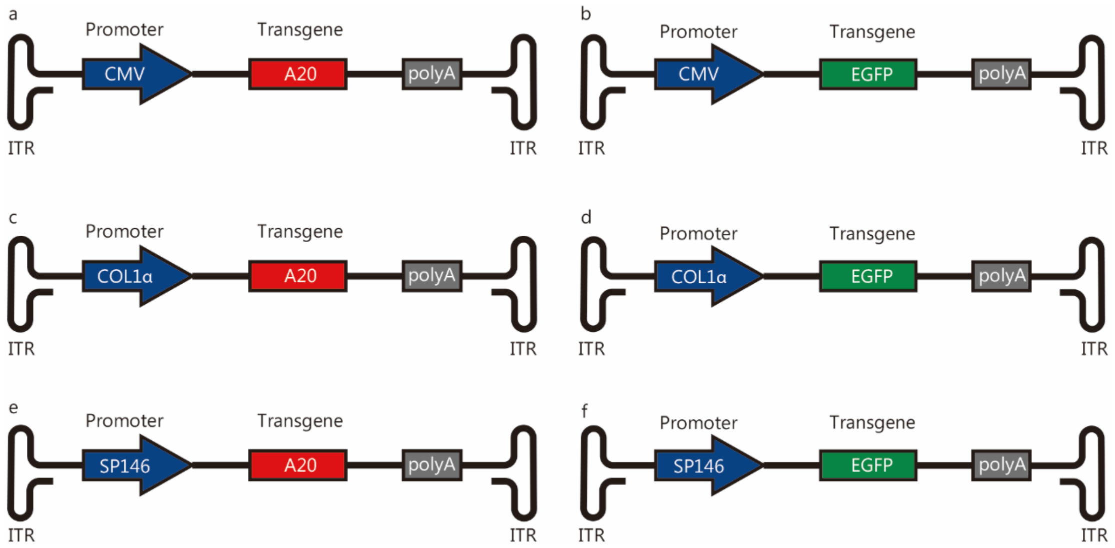

Fig. S1 Construction of rAAV expression plasmids. **a** rAAV6-CMV-A20. **b** rAAV6-CMV-EGFP. **c** rAAV6-COL1α-A20. **d** rAAV6-COL1α-EGFP. **e** rAAV6-SP146-A20. **f** rAAV6-SP146- EGFP. ITR inverted terminal repeat, COL1α collagen type I alpha 1 chain, A20 TNF-α-induced protein 3, CMV cytomegalovirus, EGFP enhanced green fluorescent protein

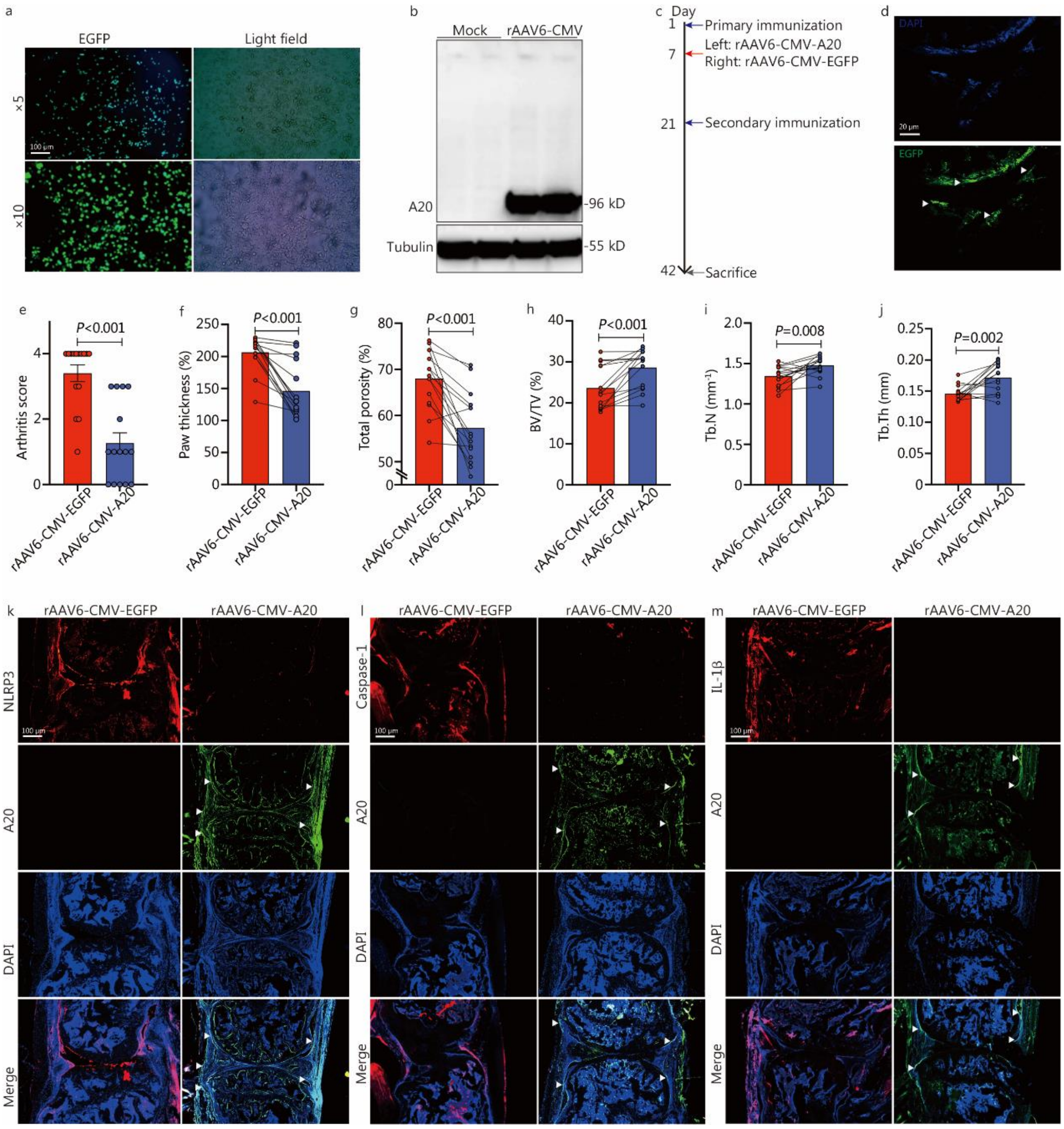

Fig. S2 Therapeutic effect of rAAV-CMV-A20. **a** Fluorescence image of 293T cells transfected with rAAV6-CMV-EGFP. **b** Western blotting of TNF-α-induced protein 3 (A20) in 293T cell lysates from cells transfected with rAAV6-CMV-A20. **c** Outline of the CIA mice model and rAAV injection (rAAV6-CMV-EGFP or rAAV6-CMV-A20). **d** Fluorescence images of EGFP in joint specimens that were injected with rAAV6-CMV-EGFP (white arrow: synovium). Clinical arthritis score (**e**) and thickness in the hind paws (**f**) decreased following injection with rAAV6-CMV-EGFP or rAAV6-CMV-A20 on the day 42nd after primary immunization (n = 15). **g-j** Quantitative analysis of total porosity, BV/TV, Tb.N, and Tb.Th (n = 15). Immunofluorescence staining of A20, NLRP3 (**k**), caspase-1 (**l**), and IL-1β (**m**) in joint specimens (white arrow: synovium). Statistical analyses were performed using the paired Wilcoxon signed rank test (**e-f**) or paired t-test (**g-j**). EGFP enhanced green fluorescent protein, CMV cytomegalovirus, A20 TNF-α-induced protein 3, NLRP3 NOD-like receptor protein 3, BV/TV bone volume-to-tissue volume ratio, Tb.N trabecular number, Tb.Th trabecular thickness

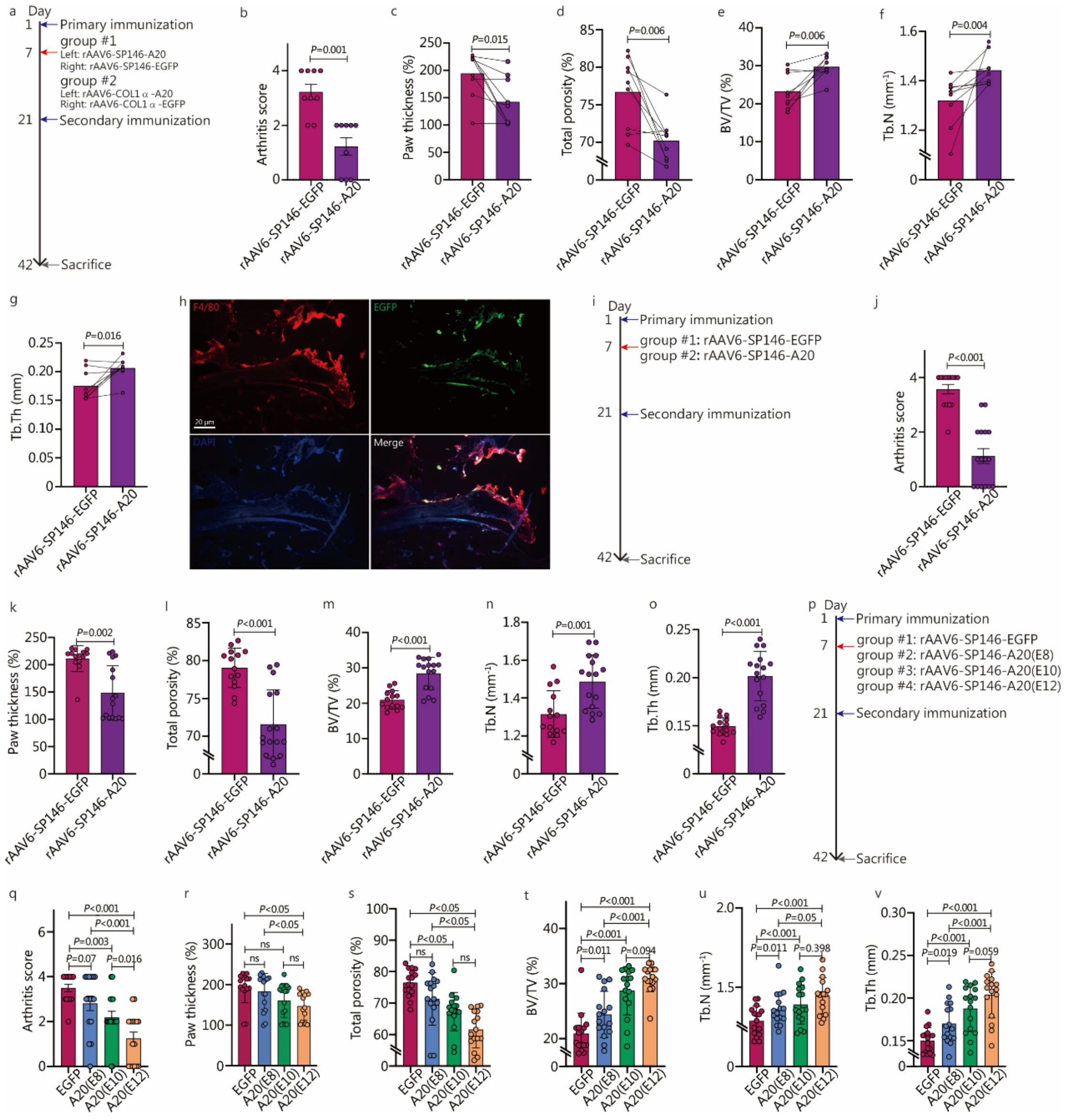

Fig. S3 Verification of the therapeutic effect of rAAV6-SP146-A20. **a** Outline of the CIA mice model and rAAV injection (rAAV6-SP146-EGFP, rAAV6-SP146-A20, rAAV6-COL1α-EGFP, rAAV6-COL1α-A20). Clinical arthritis score (**b**) and thickness of the hind paws (**c**) following injection of rAAV6-SP146-EGFP or rAAV6-SP146-A20 on the 42nd day after primary immunization (n = 9). **d-g** Quantitative analysis of total porosity, BV/TV, Tb.N, and Tb.Th (n = 9). **h** Fluorescence images of EGFP and F4/80 in joint specimens injected with rAAV6-SP146-EGFP. **i** Outline of the CIA mice model and rAAV injection (rAAV6-SP146-EGFP, rAAV6-SP146-A20). Clinical arthritis score (**j**) and thickness of the hind paws (**k**) following injection with rAAV6-SP146-EGFP or rAAV6-SP146-A20 on the day 42nd after primary immunization (n = 14 or 16). **l-o** Quantitative analysis of total porosity, BV/TV, Tb.N, and Tb.Th (n = 14 or 16). **p** Outline of the CIA mice model and rAAV injection (EGFP: 1×10^{12} vg rAAV6-SP146-EGFP; E8: 1×10^{8} vg rAAV6-SP146-A20; E10: 1×10^{10} vg rAAV6-SP146-A20; E12: 1×10^{12} vg rAAV6-SP146-A20). Clinical arthritis score (**q**) and thickness of the hind paws

(**r**) following injection with rAAV6-SP146-EGFP or rAAV6-SP146-A20 on the 42nd day after primary immunization (*n* = 16). **s-v** Quantitative analysis of total porosity, BV/TV, Tb.N and Tb.Th (*n* = 16). Statistical analyses were performed using paired *t*-tests (**c-f, h**), Paired Wilcoxon signed rank tests (**g**) and *t*-tests (**j, l-n**), Wilcoxon signed rank tests (**k, o**), ANOVA followed by post hoc Student-Newman-Keuls pairwise comparisons (**q, s, u-v**) or Kruskal-Wallis one-way analysis of variance on ranks tests followed by post hoc Tukey's comparisons (**r, t**). CIA collagen-induced arthritis, EGFP enhanced green fluorescent protein, COL1α collagen type I alpha 1 chain, CMV cytomegalovirus, A20 TNF-α-induced protein 3, BV/TV bone volume-to-tissue volume ratio, Tb.N trabecular number, Tb.Th trabecular thickness, ns non-significant

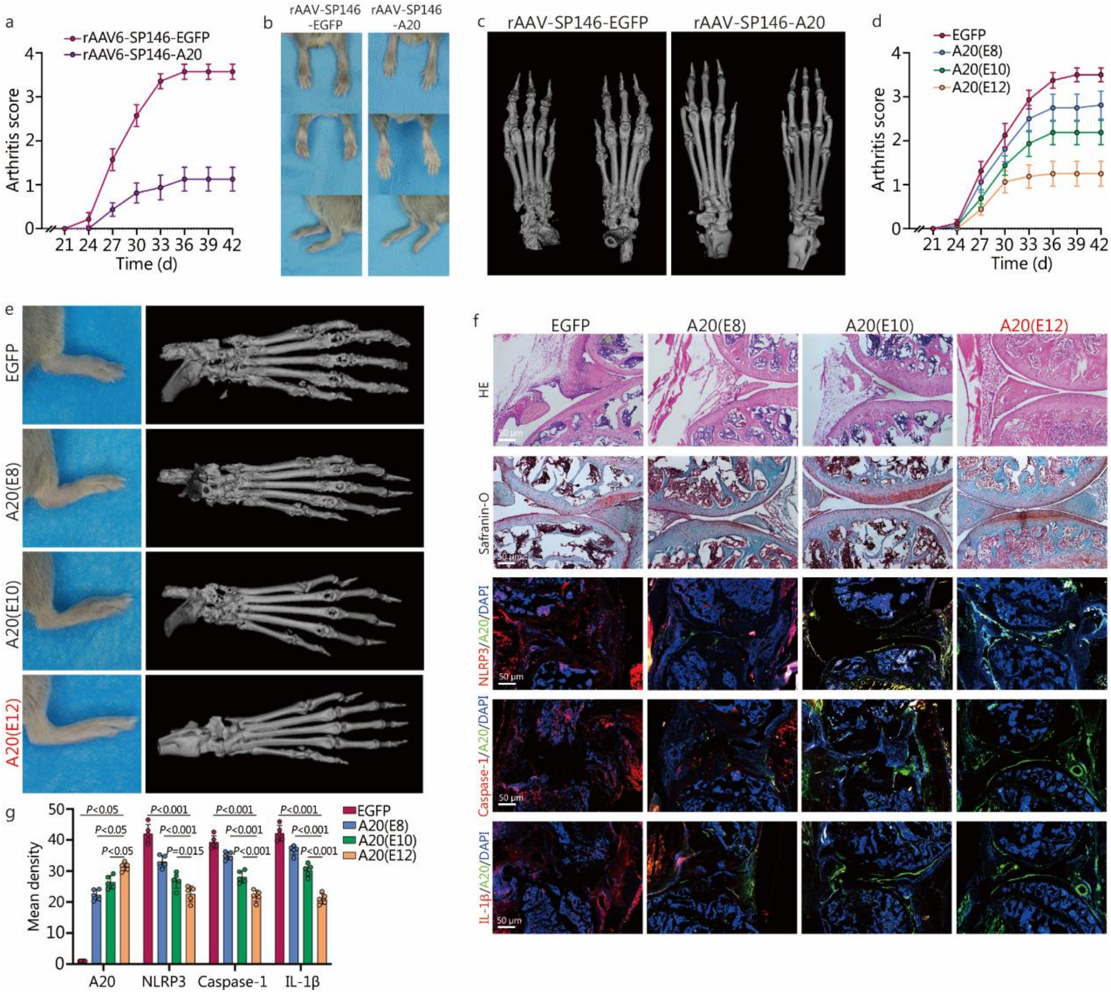

Fig. S4 rAAV6-SP146-A20 inhibits NLRP3 inflammation in CIA mice. **a** Clinical arthritis score of the hind paws injected with rAAV6-SP146-EGFP or rAAV6-SP146-A20. **b** Hind paws of CIA mice injected with rAAV6-SP146-EGFP or rAAV6-SP146-A20 on the day 42nd after primary immunization. **c** Micro-CT 3D reconstruction image of the hind paws. **d** Clinical arthritis score of hind paws injected with rAAV6-SP146-EGFP (1×10^{12} vg), rAAV6-SP146-A20 (1×10^{8} vg), rAAV6-SP146-A20 (1×10^{10} vg), or rAAV6-SP146-A20 (1×10^{12} vg). **e** Hind paws of CIA mice and micro-CT 3D reconstruction image of the hind paws. **f** HE and Safranin-O staining of joint specimens. Immunofluorescence staining of A20, NLRP3, caspase-1, and IL-1β in joint specimens. **g** Quantification of A20, NLRP3, caspase-1, and IL-1β using the mean density (integrated density/specimen area) in joint specimens (n = 5). Statistical analyses were performed using ANOVA followed by post hoc Student-Newman-Keuls pairwise comparisons (NLRP3, caspase-1, and IL-1β) or Kruskal-Wallis one-way analysis of variance on ranks followed by post hoc Student-Newman-Keuls pairwise comparisons (A20). CIA collagen-induced arthritis, EGFP enhanced green fluorescent protein, A20 TNF-α-induced protein 3, NLRP3 NOD-like receptor protein 3
